# MicroRNA-989 controls *Aedes albopictus* pupal-adult transition process by influencing cuticle chitin metabolism in pupae

**DOI:** 10.1186/s13071-023-05976-x

**Published:** 2023-11-02

**Authors:** Ruiling Zhang, Wenjuan Liu, Jingwen Fu, Zhong Zhang

**Affiliations:** 1 Shandong Provincial Hospital Affiliated to Shandong First Medical University, Jinan, 250000 China; 2 School of Clinical and Basic Medical Science, Shandong Academy of Medical Sciences), Shandong First Medical University, Jinan, 250117 China; 3 School of Laboratory Animal (Shandong Laboratory Animal Center), Shandong Academy of Medical Sciences), Shandong First Medical University, Jinan, 250117 China

**Keywords:** miRNA, Chitin metabolism, Mosquito, Chitin synthases, Chitinases

## Abstract

**Background:**

*Aedes albopictus* is a vector of numerous devastating arboviruses and places heavy burdens on global public health. Chitin is one of the important components of cuticles and targeting chitin metabolism is a promising strategy for preventing mosquito dispersal and mosquito-borne diseases. Increasing evidence suggests that microRNAs (miRNAs) play crucial roles in various physiological processes of insects.

**Methods:**

A previous analysis suggested that the microRNA miR-989 is potentially involved in chitin metabolism in *Ae. albopictus* pupae. In the present study, we found that the expression level of miR-989 was significantly overexpressed after injection of agomir. A dual-luciferase assay was used to determine the direct target of miR-989. Survival rate, eclosion rate and malformation rate were statistically analyzed to evaluate the potential effect of miR-989. Hematoxylin–eosin staining and chitin staining were used to evaluate the microstructural changes in the cuticles of *Ae. albopictus* pupae.

**Results:**

Overexpression of miR-989 resulted in a significantly reduced survival rate and eclosion rate of pupae and an elevated malformation rate of adults. The results suggested that miR-989 acted as a regulator of chitin metabolism in *Ae. albopictus* pupae by affecting the transcript levels of the *Ae. albopictus* genes encoding chitin synthase 1 (*AaCHS*1) and chitinase 10 (*AaCht*10). The altered expression levels of the two chitin metabolism-related enzymes (CHS1 and Cht10, respectively) caused the structural changes in cuticles and further affected the pupal-adult transition process of *Ae. albopictus*. XM_029863591.1 was proven to be the target gene of miR-989 and displayed similar effects on pupae as miR-989.

**Conclusions:**

The microRNA miR-989 was found to be essential for chitin metabolism in old and new cuticles of *Ae. albopictus* pupae. The results of the current study suggested that miR-989 could be used as a potential target to control *Ae*. *albopictus*.

**Graphical Abstract:**

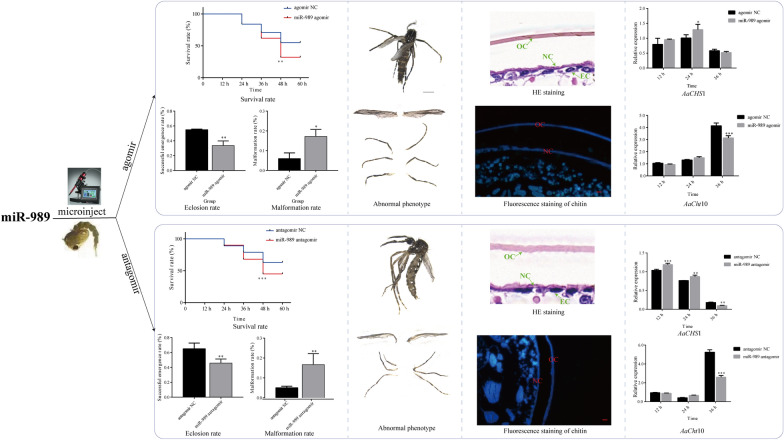

**Supplementary Information:**

The online version contains supplementary material available at 10.1186/s13071-023-05976-x.

## Background

The Asian tiger mosquito, *Aedes albopictus*, is an important vector of dengue virus, chikungunya virus, Zika virus and other arboviruses. As one of the most invasive mosquito species [1], *Ae*. *albopictus* poses a serious threat to global public health [2]. It is estimated there are 96 million cases of dengue annually, with more than 3.9 billion people in over 128 countries at risk contracting dengue fever [3]. In the absence of prophylactic drugs and effective vaccines, the prevention and control of mosquito-borne diseases are heavily reliant on eliminating vector populations. Due to the long-term and large-scale use of insecticides, severe resistance to most of the WHO-approved public health insecticides has been detected in mosquito populations worldwide [4, 5]. This situation demonstrates that innovative vector control measures that can complement insecticides are urgently needed.

Cuticles serve as barriers protecting the insects from physical and chemical injuries, dehydration, pathogen infections and predation [6]. As the major component of cuticles, chitin is composed of β−1,4 linked N-acetylglucosamines [7] and plays not only an important role in the rigidity of the cuticle but also functions as the attachment matrix for cuticular proteins [7]. Despite providing physical support and protection, cuticles restrict insect growth and development and, therefore, insects have adopted the strategy of periodic molting to cope with the constraint of the cuticle. During each molt, insects synthesize a new cuticle and partially digest and shed the old cuticle from the previous developmental stage as the exuvium [7]; chitins are therefore required at each developmental stage of insects [6]. Abnormal chitin metabolism results in dysfunction of cuticular structures and hinders the growth and development of insects [7]. Chitin is one of the necessary survival structures of insects but it is absent in plants and vertebrates [8], indicating its potential as an excellent target for developing a safe and effective control strategy for mosquitoes.

Chitin metabolism is a complex and multistep cascade involving a series of enzymes, of which chitin synthases (CHS) and chitinases (Cht) are the two important enzyme families. Two chitin synthases (CHS1 and CHS2) have been characterized in insect genomes [9]. CHS1 is responsible for chitin synthesis in tissues such as the epidermis, trachea and salivary gland; CHS2 is specifically expressed in the midgut and is involved in chitin biosynthesis of the peritrophic matrix (PM) [9–12]. However, the number, enzymatic properties, and expression patterns of insect chitinases differ substantially between species [6, 13].

microRNAs (miRNAs) are a class of small noncoding RNAs (18–25 nucleotides) that have emerged as key posttranscriptional regulators of gene expression. Generally, through binding to miRNA response elements (MREs), which are located on the 3’-untranslated regions (3’UTRs) or the coding sequences (CDSs) of target messenger RNAs (mRNAs), miRNAs function as fine-tuners to prevent translational events through mRNA degradation or translational repression [14]. Accumulating evidence has been shown that miRNAs participate in the regulation of several physiological processes in insects [15]. For example, miR-8 was proven to be involved in regulating the reproductive processes of *Aedes aegypti* [16], and miR-275 was found to be indispensable for blood digestion and egg development in *Ae*. *aegypti* [17]. In addition, several distinct miRNAs have been proven to be involved in chitin metabolism. miR-8-5p and miR-2a-3p can tune the chitin biosynthesis pathways in *Nilaparvata lugens* [18]; miR-71 and miR-263 regulate *Locusta migratoria* molting by targeting *LmCHS*1 and *LmCHT*10, respectively [19]; and miR-282-5p can regulate larval molting of *Bombyx mori* [20].

The efficiency of miRNAs in chitin metabolism has prompted researchers to exploit miRNA-based approaches for insect control [21–24]. For *Ae. albopictus*, the regulatory mechanism of miRNAs in chitin remains elusive, and more candidate miRNAs need to be identified and knowledge gaps bridged regarding how miRNAs regulate chitin metabolism in order to utilize them efficiently. A previous bioinformatics study with *Ae. albopictus* found that the microRNA miR-989 was potentially involved in chitin metabolism in pupae [25]. In the present study, the efficiency of miR-989 in influencing chitin metabolism was evaluated based on phenotype observation, statistical analysis of three indicators (including survival rate, eclosion rate, and malformation rate), hematoxylin–eosin (HE) staining and fluorescence staining of chitin after overexpression and suppression of miR-989. Additionally, the relationships between the miR-989 and the *Ae. albopictus* genes encoding chitin synthase 1 (CHS1; *AaCHS*1) and chitinase 10 (Cht10; *AaCht*10) were exploited to decode the molecular mechanisms underlying chitin metabolism.

## Materials and methods

### Sampling preparation

The *Ae. albopictus* strain used in this study was collected in Shandong Province, China. In the laboratory, the field-collected mosquitoes were reared under a daily photoperiod of 14:10 h (light:dark) at 27 ± 1 °C and 65% relative humidity. Pupae were collected at 12–18 h after pupation for microinjection. Dissections of pupae were performed under a stereoscopic microscope (Leica Microsystems, Wetzlar, German) to obtain the cephalothorax, epidermis, midgut and Malpighian tube. All samples were collected with three biological replicates, flash frozen in liquid nitrogen immediately following collection and then stored at − 80 °C until RNA isolation.

### RNA extraction, complementary DNA synthesis and quantitative real-time PCR analysis

Total RNA was extracted using RNA Isolater Total RNA Extraction Reagent (Vazyme, Nanjing, China) according to the manufacturer’s instructions. The quality and quantity of RNA were determined by electrophoresis in 2% agarose gels and spectrophotometry (ScanDrop spectrophotometer; Analytik Jena, Jena, Germany).

The first-strand complementary DNA (cDNA) of miRNA and mRNA was synthesized by a miRNA First-Strand cDNA Synthesis Kit (by stem‒loop) (Vazyme) and HiScript III RT Supermix for quantitative PCR (+ gDNA wiper) (Vazyme) using 2 μg of total RNA as template. Quantitative real-time PCRs (qRT‒PCR) of miRNA and mRNA were performed using miRNA Universal SYBR qPCR Master Mix (Vazyme) and ChamQ Universal SYBR qPCR Master Mix (Vazyme) according to the manufacturer’s protocols. The reaction system of qRT‒PCR included 2.0 μl of cDNA, 0.4 μl forward and reverse primer (10 μM), 10 μl 2× miRNA Universal SYBR qPCR Master Mix for miRNA or 2× ChamQ Universal SYBR qPCR for mRNA, with 10 μl RNase-free water was added. The primers used in this study are shown in Additional file 1: Table S1.

The qRT‒PCR cycling reaction consisted of an initial denaturation at 95 °C for 5 min, followed by 40 cycles at 95 °C for 10 s, 60 °C for 30 s and 95 °C for 10 s. All qRT–PCRs were performed in an ABI7500 Real-Time PCR System (Thermo Fisher Scientific, Waltham, MA, USA). All experiments were performed in three biological and three technical replicates. The relative expression was measured using the 2^−ΔΔCT^ method [26], and the expression levels of miRNAs and mRNAs were normalized against U6 and β-actin, respectively.

### miRNA agomir treatment

The miR-989 agomir was synthesized by GenePharma Biotechnology (Shanghai, China) (Additional file 1: Table S2). Pupae were anesthetized on ice, and the agomir was microinjected into the hemolymph through the dorsal cuticle between the thorax and abdomen [27] using a Nanoject III injector (Drummond Scientific, Broomall, PA, USA) at a dose of 50 pmol. The equivalent volume of agomir negative control (agomir NC) was also injected into the hemolymph. Pupae were collected for further analysis at 12, 24 and 36 h post injection. All collected samples used for qRT–PCR were flash frozen in liquid nitrogen immediately following collection and then stored at − 80 °C.

### Prediction of microRNA targets

RNAhybrid [28] and miRanda [29] were used to predict microRNA targets. The final target was intersected with the gene sets identified by two software programs. The minimum free energy (MFE) of each interaction was calculated, and the smaller the value of MFE, the more stable the interaction between the microRNA and its target.

### Luciferase reporter assay

For the luciferase reporter analysis, the binding site of miR-989 and its target was used as the insert sequence and clustered into the pmirGLO vector (Promega, Madison, WI, USA). XM_029863591.1 is a chitin-binding protein. Wild-type (WT) pmirGLO_XM_029863591.1_WT and mutant (Mut) pmirGLO_XM_029863591.1_Mut plasmids were designed and synthesized. In the mutant plasmid, the inserted sequence that included regions bound to the seed sequence of miR-989 was mutated by using site mutation. HEK293T cells were first cultured in an incubator at 37 °C and 5% CO_2_ and then transfected with pmirGLO_XM_029863591.1 plasmids (WT or Mut) and agomir or agomir NC of miR-989 using Lipofectamine 2000 transfection reagent (Thermo Fisher Scientific) following the manufacturer’s instructions. Transfected cells were cultured in an incubator for 20 min and then transferred into new culture medium containing 10% fetal bovine serum. Luciferase activities were measured at 24 h after transfection using a Dual Luciferase Reporter Assay Kit (Vazyme). *Firefly* luciferase was used to normalize *Renilla* luciferase expression. All experiments were performed in triplicate and repeated three times.

### RNA interference-mediated gene silencing

The specific primers containing the T7 polymerase promoter sequence (TAATACGACTCACTATAGGG) were designed using E-RNAi-v3.2 (http://www.dkfz.de/signaling/e-rnai3/idseq.php). The enhanced green fluorescent protein (*eGFP*) gene (GenBank accession number: DQ768212.1) was used as control. Then, cDNA fragments were generated by PCR using primers fused with T7 promoter sequences. Double-stranded RNAs (dsRNAs) were synthesized using a T7 RNAi transcription kit (Vazyme) according to the manufacturer's instructions.

For the knockdown of gene expression, 750 ng dsRNA was injected into the pupae using a Nanoject III injector (Drummond Scientific). Pupae injected with equivalent volumes of ds*eGFP* were used as controls. Samples were collected at 12, 24 and 36 h post injection, and the efficiency of RNA interference (RNAi) was evaluated by qRT–PCR. All collected samples were flash frozen in liquid nitrogen immediately following collection and then stored at − 80 °C.

### Phenotypic observation

At 12 h post infection with agomir/dsRNA, living pupae were chosen for phenotypic observations (30 pupae in each group). The status of pupae was recorded every 12 h until all living pupae were eclosed and then statistical analyses were performed of the survival rate, eclosion rate and malformation rate. All experiments were performed with three biological replications. The abnormal phenotypes were visualized under a stereomicroscope and photographed using Leica Application Suite V4 (Leica Microsystems).

### Hematoxylin–eosin staining

At 36 h after the injection of agomir/dsRNA, the chitin structures of pupae were examined using hematoxylin–eosin (HE) staining. Cuticles were dissected from the middle of the third abdomen of pupae and fixed in 4% paraformaldehyde. Paraffin sections (thickness: 4 μm) were prepared after dehydration, transparency, embedding, and staining with HE as described previously [30]. The images were then collected using the Pannoramic 250 digital scanner (3DHISTECH, Budapest, Hungary).

### Fluorescence staining of chitin

Paraffin sections were obtained from the same samples used for HE staining. Sections were dewaxed using xylene (2 times, 10 min each time), dehydrated through an ethanol series (100%, 95%, 90%, 85% and 70% ethanol successively, 5 min each time) and washed in distilled water (5 min). After dewaxing, the sections were washed using phosphate-buffered saline (PBS) three times (5 min each time) and then incubated with Calcofluor White (CFW) (1 mg/ml; Sigma–Aldrich, St. Louis, MO USA) for 5 min. Finally, the sections were washed using PBS three times (5 min each time), and images were captured using a fluorescence microscope (model NIB410-FL, Yongxin, Ningbo, China) with a 405 nm laser.

### Statistical analysis

The statistical analysis was performed using GraphPad Prism software version 8.0 (GraphPad Software, San Diego, CA, USA), and values are represented as the mean ± standard error (SE). The statistical significance was determined by Student’s t-test for unpaired comparisons between two different groups. Statistically significant differences are denoted by asterisks (*P < 0.05), (**P < 0.01) and (***P < 0.001). The log-rank test and Mantel–Cox test in the Kaplan–Meier method were used to determine the survival rate.

## Results

### Identification of miR-989 target

**Fig. 1:**
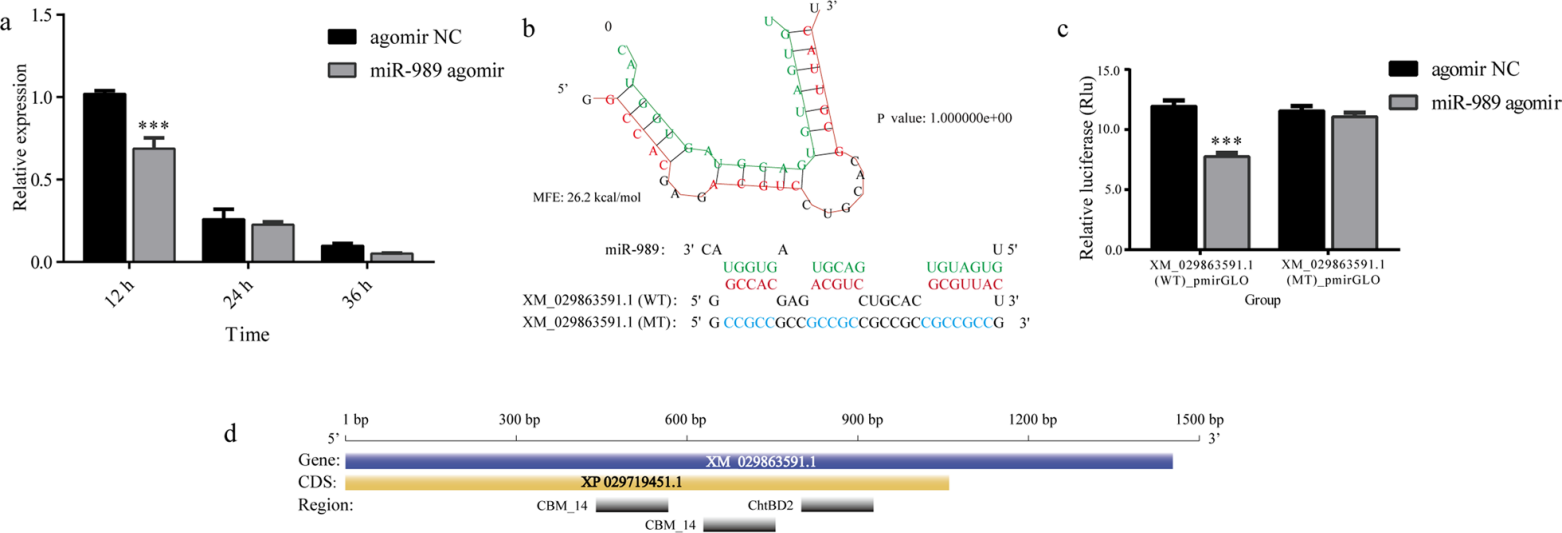
Interaction between miR-989 and XM_029863591.1. **a** Expression level of XM_029863591.1 after overexpression of miR-989. **b** The predication of binding site of miR-989 and XM_029863591.1 *WT* wild type, *MT* mutant type; **c** Luciferase activity in HEK293T cells transfected with different vectors; **d** Schematic diagram of XM_029863591.1. Asterisks indicate a significant difference at **P < 0.01 and ***P < 0.001. CDS, Coding sequence; MFE, minimum free energy; MT, mutant type; NC, negative control; WT, wild type

According to the results predicted with RNAhybrid and miRanda, XM_029878882.1 and XM_029863591.1 were potential targets of miR-989. The results of qRT–PCR suggested that the expression level of XM_029878882.1 had no relationship with miR-989 (Additional file 2: Fig. S1), while the expression level of XM_029863591.1 showed an obvious negative correlation with miR-989 (Fig. 1a). Injection of miR-989 agomir significantly reduced the transcript level of XM_029863591.1 at 12 h post injection compared with the control group (agomir NC) (*P* < 0.001) (Fig. 1a). The binding site of miR-989 was located in the CDS region (639 bp-667 bp) of XM_029863591.1, with an MFE value of - 26.2 kcal/mol (Fig. 1b). The interactions between miR-989 and XM_029863591.1 were further verified using a fluorescent luciferase reporter assay in vitro. The luciferase activity was significantly lower than in the control group (*P* < 0.001) when HEK293T cells were cotransfected with the recombinant WT plasmid XM_029863591.1 (i.e. pmirGLO_XM_029863591.1_WT) with miR-989 agomir, while no obvious change in luciferase activity was observed when the seed sequence of XM_029863591.1 was mutated (i.e. pmirGLO_XM_029863591.1_Mut) (Fig. 1c).

The full length of XM_029863591.1 is 1320 bp, encoding 354 amino acids (aa). According to the annotation information in the NCBI, XM_029863591.1 is a chitin-binding domain protein CBD-1-like (gene symbol: LOC115261703), including one protein-coding DNA sequence that consists of two chitin-binding peritrophin-A domains (CBM_14) and one chitin-binding domain type 2 (ChtBD2) (Fig. 1d).

### Expression patterns of miR-989 and XM_029863591.1

**Fig. 2:**
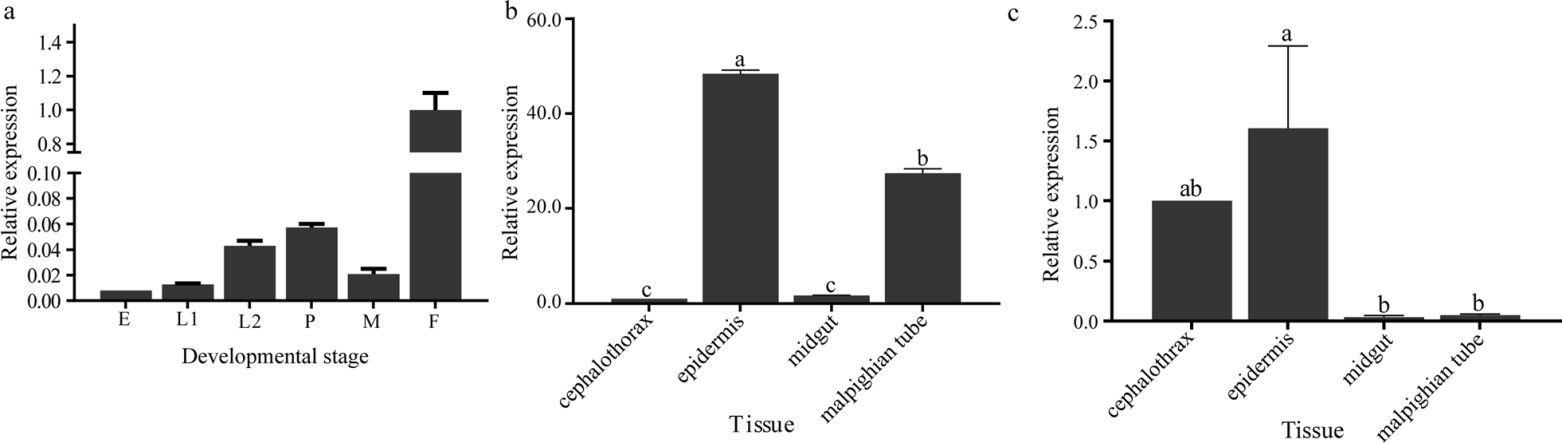
Expression of miR-989 and XM_029863591.1. **a** Expression of miR-989 at different developmental stages. **b** Expression of miR-989 in different tissues of pupa. **c** Expression of XM_029863591.1 in different tissues of the pupa. Lowercase letters above bars indicate statistically significant differences between different groups. E, Egg; F, female; L, larval; P, pupa

The results of the temporal expression pattern indicated that the expression of miR-989 varied with the developmental stages of *Ae. albopictus*. The qRT–PCR results showed that the expression level of miR-989 peaked in females and was second highest in the pupal stage (Fig. 2a). Among the four tissues of pupae studied, the highest expression level of miR-989 was detected in the epidermis, followed by the Malpighian tube, and the lowest level was measured in the cephalothorax (Fig. 2b). The highest transcript level of XM_029863591.1 was detected in the epidermis (Fig. 2c).

### Effects of miR-989 and XM_029863591.1 on pupa development

#### miR-989

**Fig. 3:**
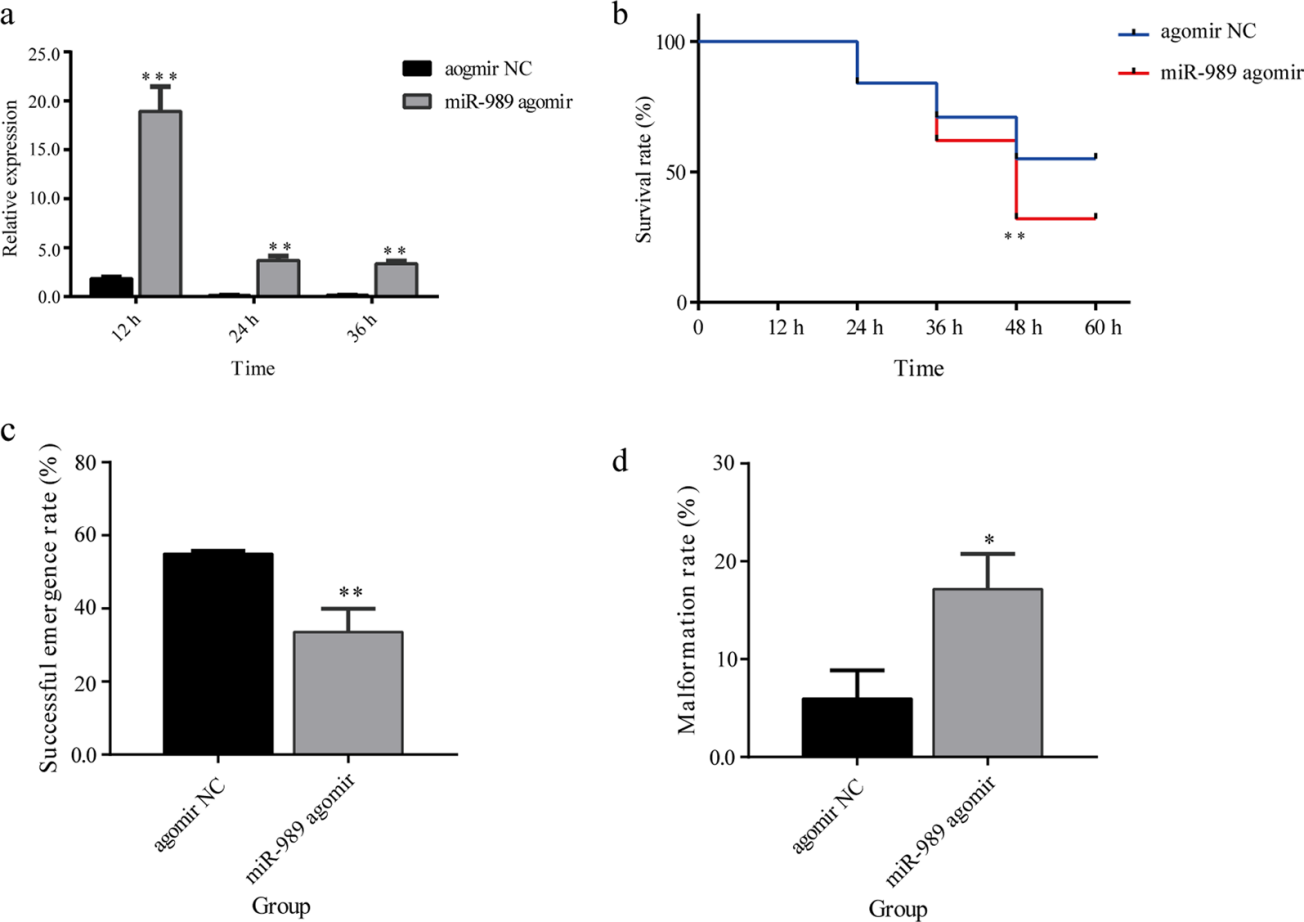
Effects of miR-989. **a** The efficiency of overexpression of miR-989. **b** Survival rate of pupae after overexpression of miR-989. **c** Eclosion rate of pupae after overexpression of miR-989. **d** Malformation rate after overexpression of miR-989. Asterisks indicate statistically significant differences at *P < 0.05, **P < 0.01 and ***P < 0.001. NC, Negative control

qRT–PCR was performed to evaluate the abundance of miR-989 after the microinjection of agomir. At 12, 24 and 36 h after injection, the expression level of miR-989 was significantly increased in pupae treated with agomir compared with the control group pupae (*P* < 0.01) (Fig. 3a).

**Fig. 4:**
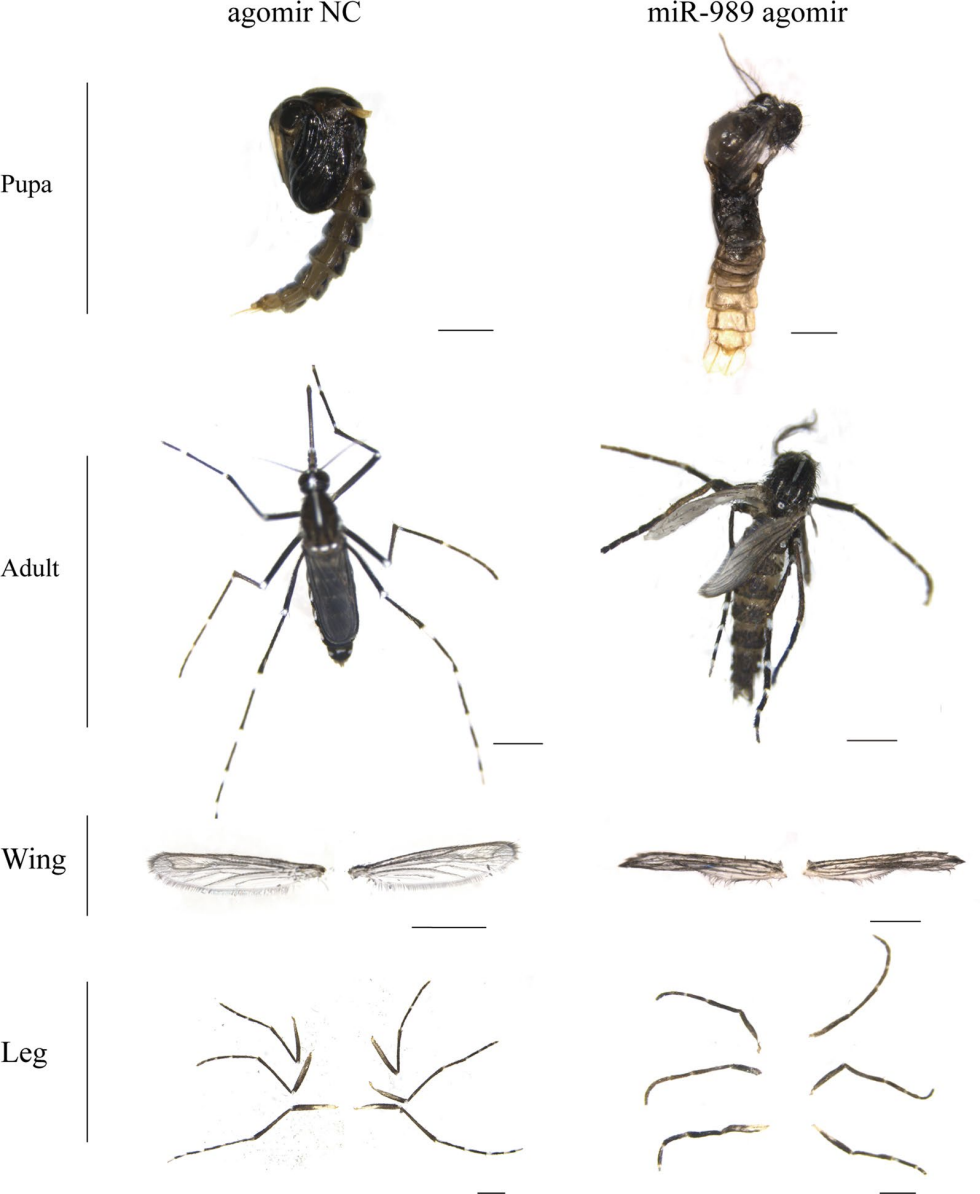
Phenotypes of pupae and adults after overexpression of miR-989. Scale bar: 1 mm. NC, Negative control

Pupae treated with agomir were screened for phenotypic changes to assess the functions of miR-989. Phenotypic observation suggested that overexpression of miR-989 could significantly affect the development of pupae. At 48 h after injection, the survival rate in the agomir-treated group was 33.54%, which was significantly lower than that in the control groups (*P* < 0.01) (Fig. 3b). Many pupae in the agomir-treated group failed to detach the old cuticle and died without complete emergence (Fig. 4). In addition, all living pupae were eclosed at 48 h after injection; thus, the eclosion rate (33.54%) at this time was equal to the survival rate, which was significantly lower than that of the control group (*P* < 0.01) (Fig. 3c).

The malformation rate reached 17.17% after overexpression of miR-989, which was significantly higher than that in the control group (*P* < 0.05) (Fig. 3d). The abnormal phenotypes caused by overexpression of miR-989 included malformed wing and malformed leg (Fig. 4). Specifically, some successfully emerged adults had shrinking wings or curled legs, which caused adults to be unable to fly and to die within a short time.

#### XM_029863591.1

**Fig. 5:**
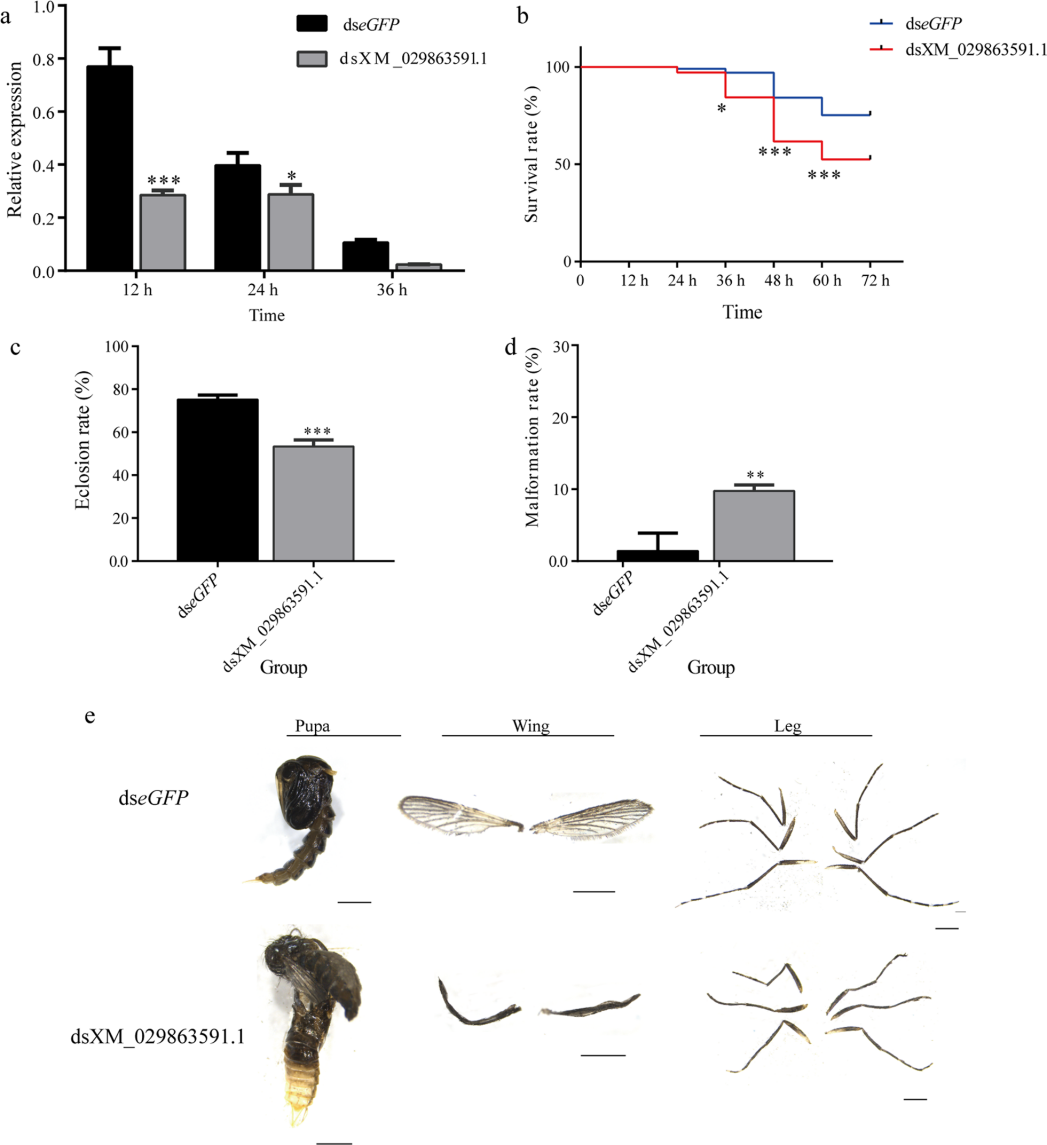
Effects of XM_029863591.1. **a** The efficiency of RNAi of XM_029863591.1. **b** Survival rate of pupae after RNAi of XM_029863591.1. **c** Eclosion rate of pupae after RNAi of XM_029863591.1. **d** Malformation rate after RNAi of XM_029863591.1. **e** Phenotypes of pupae and adults after RNAi of XM_029863591.1. Asterisks indicate statistically significant differences at *P < 0.05, **P < 0.01 and ***P < 0.001. ds, Double-stranded;* eGFP*, enhanced green fluorescent protein gene (control); RNAi, RNA interference

The efficiency of RNAi was evaluated using qRT–PCR. The results showed that the expression level of XM_029863591.1 was significantly decreased with RNAi compared with that in the control group at 12 h and 24 h after injection of dsXM_029863591.1 (*P* < 0.05) (Fig. 5a).

After silencing XM_029863591.1, the survival rate of the treated group rapidly decreased and was reduced to 84.33% at 36 h, 62.36% at 48 h and 53.41% at 60 h post injection, which were significantly lower survival times than those of the control groups (*P* < 0.05) (Fig. 5b). All surviving pupae emerged at 60 h after injection, and the eclosion rate of the dsXM_029863591.1 treated group (53.41%) was significantly lower than that of the control group (*P* < 0.001) (Fig. 5c). The malformation rate in the dsXM_029863591.1-treated group was 9.76%, which was much higher than that in the control group (*P* < 0.01) (Fig. 5d). The malformation phenotypes caused by injection of dsXM_029863591.1 were similar to those found in overexpression of miR-989. Some adults emerged with wrinkled wings or malformed legs (Fig. 5e).

### Effects of miR-989 and XM_029863591.1 on cuticle structure of pupae

**Fig. 6:**
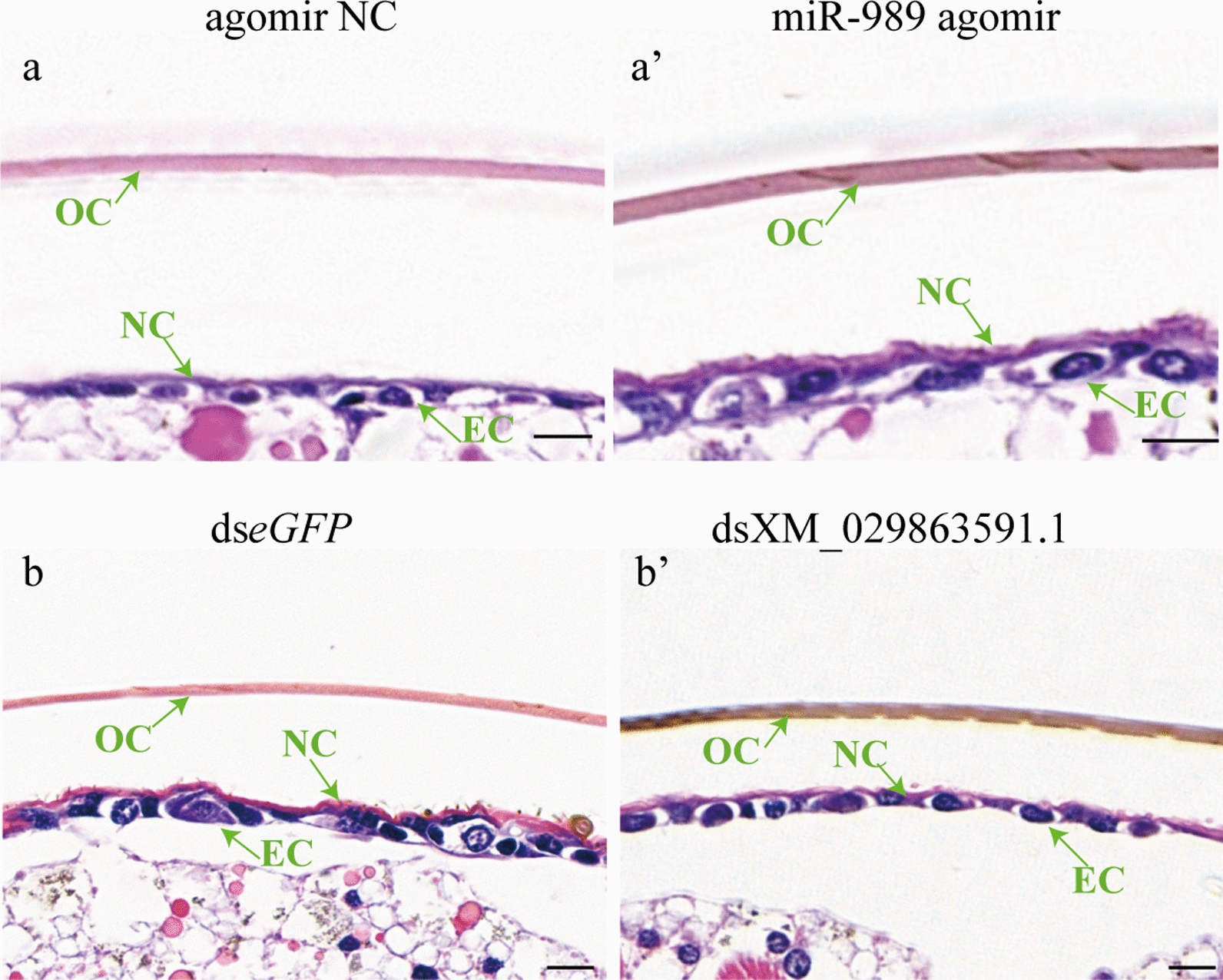
Hematoxylin–eosin staining of epidermis of pupae.** a**,** a′** Effects of agomir NC (**a**) and miR-989 agomir (**a**’).** b**,** b′** Effects of ds*eGFP* (**b**) and dsXM_029863591.1 (**b**’). Scale bar: 10 μm. Agomir NC, Agomir negative control; ds, double-stranded; EC, epidermal cell;* eGFP*, enhanced green fluorescent protein gene (control), NC, new cuticle; OC, old cuticle

Staining with HE was performed to verify the effects of miR-989 and XM_029863591.1 on the structure of cuticles. The results showed that both the old (3.36 ± 0.21 μm) and new (2.16 ± 0.39 μm) cuticles in the miR-989- and XM_029863591.1-treated groups were thicker than those in the control groups (old: 2.91 ± 0.06 μm; new: 1.28 ± 0.22 μm) in response to overexpression of miR-989 (Fig. 6a, a’) (*P* < 0.01). In addition, HE staining revealed that in the miR-989 agomir-treated group, the size of epidermal cells was irregular and that the thickness of the newly formed cuticle was uneven compared to those of the control group, respectively (Fig. 6a, a’).

After knockdown of XM_029863591.1, the old cuticle (3.51 ± 0.13 μm) of the treated group was much thicker than that of the control group (2.55 ± 0.14 μm) (*P* < 0.001); however, there was no significant difference in the thickness of the new cuticle between the treated and control groups (Fig. 6b, b’). Similarly, an irregular size of the epidermal cells and uneven thickness of newly formed cuticle were detected in the dsXM_029863591.1-treated group compared with those in the ds*eGFP*-treated group according to HE staining (Fig. 6b, b’).

**Fig. 7:**
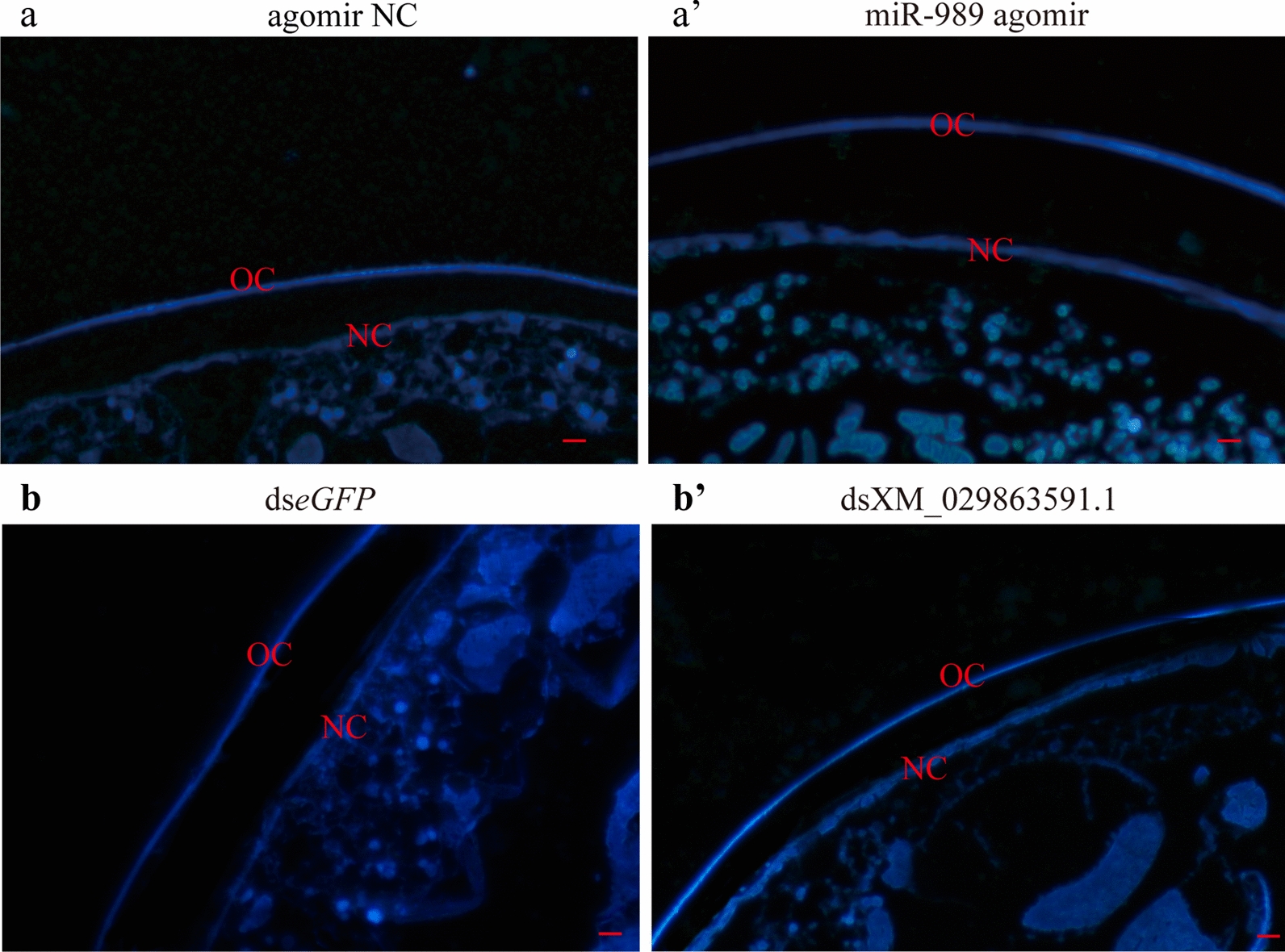
Fluorescence staining of chitin.** a**,** a′** Effects of miR-989 on chitin of the new and old cuticle after injection of agomir NC (**a**) and miR-989 agomir (**a**’).** b**,** b′** Effects of XM_029863591.1 on chitin of the new and old cuticle after injection of ds*eGFP* (**b**) and dsXM_029863591.1 (**b**’). Scale bar, 10 μm. Agomir NC, Agomir negative control; ds, double-stranded; *eGFP*, enhanced green fluorescent protein gene (control), NC, new cuticle; OC, old cuticle

To verify the changed thickness of old and new cuticles revealed by HE staining, fluorescence staining of chitin using CFW was performed to confirm the changes in chitin structure. As shown in Fig. 7, both the old and new cuticles in the agomir-treated group presented much stronger fluorescence signals than those in the control group (Fig. 7a, a’), indicating the accumulation of chitin. In addition, an enhanced fluorescence signal of chitin was also detected in the old cuticle of the dsXM_029863591.1-treated group, while no significant difference was observed in the new cuticle between the treated and control groups (Fig. 7b, b’).

### Effects of miR-989 and XM_029863591.1 on the expression patterns of *AaCHS*1 and *AaCht*10

*AaCHS*1 is specifically expressed in epidermal tissues [8–11], and *AaCht*10 was found to be highly expressed in the epidermis of *Ae*. *albopictus* [30]. Considering the highly expressed pattern of miR-989 in the epidermis, *AaCHS*1 and *AaCht*10 were used as representative of the effects of miR-989 on chitin metabolism-related enzymes.

**Fig. 8:**
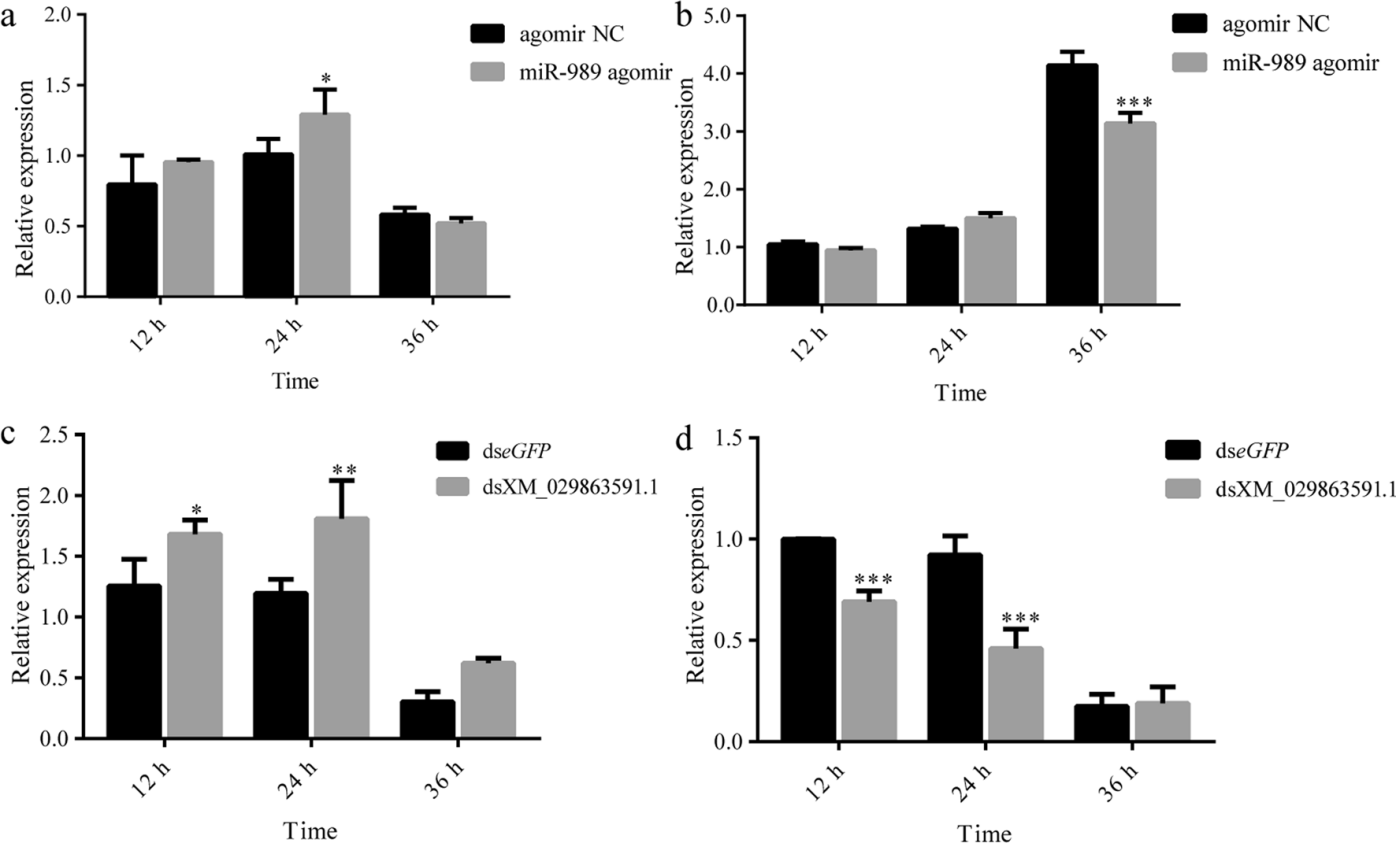
Effects of miR-989 and XM_029863591.1 on the expression of *AaCHS*1 and *AaCht*10. **a** Expression of *AaCHS*1 after overexpression of miR-989. **b** Expression of *AaCht*10 after overexpression of miR-989. **c** Expression of *AaCHS*1 after RNAi of XM_029863591.1. **d** Expression of *AaCht*10 after RNAi of XM_029863591.1. Asterisks indicate significant differences at *P < 0.05, **P < 0.01 and ***P < 0.001. *AaCHS*1 *Ae. albopictus* gene encoding chitin synthase 1 (CHS1); *AaCht*10, *Ae. albopictus* gene encoding chitinase 10 (Cht10); ds, double-stranded; *eGFP*, enhanced green fluorescent protein gene (control); NC, negative control

Along with the overexpression of miR-989, compared to the control group, the expression level of *AaCHS*1 was significantly increased at 24 h (*P* < 0.05) (Fig. 8a); the expression level of *AaCht*10 was significantly decreased at 36 h (*P* < 0.001) (Fig. 8b). Similar results were also detected after injection of dsRNA of XM_029863591.1; the expression level of *AaCHS*1 was significantly increased at 12 h and 24 h (*P* < 0.05) (Fig. 8c), while the expression levels of *AaCht*10 were significantly decreased at 12 h and 24 h (*P* < 0.001) (Fig. 8d).

## Discussion

Molting is an essential process for insect development, and chitin metabolism is crucial to the successful molting and metamorphosis of insects [31, 32]. The growth and development of insects will be hindered once chitin biosynthesis in the new cuticle or the degradation of the old cuticle is interrupted [6]. Therefore, the key genes that manipulate insect chitin metabolism can be used as candidate targets for insect control [7, 33, 34].

It has been reported that miR-989 is highly expressed in female *Anopheles gambiae* [35], *Anopheles stephensi* [36] and *Aedes aegypti* [37] compared to adult males. The predominant expression in the ovaries suggests that miR-989 is associated with reproduction in female mosquitoes [37]. Similarly, in the present study we found that miR-989 was highly expressed in female *Ae. albopictus*, as in other mosquitoes (Fig. 2a). Nevertheless, the results of the current study suggest that miR-989 can influence chitin metabolism in *Ae*. *albopictus* pupae. The negatively regulated relationships between miR-989 and XM_029863591.1 (Fig. 1a), as well as the significantly reduced *Renilla* luciferase activity when cotransfected with wild-type plasmid XM_029863591.1 with miR-989 agomir in a dual luciferase reporter assay, confirmed that XM_029863591.1 is a target of miR-989 in vitro and in vivo (Fig. 1c). Overexpression of miR-989 in *Ae*. *albopictus* pupae resulted in highly increased failure rate in the pupal–adult transition, which manifested as a significantly decreased survival rate and eclosion rate in pupae, as well as an obviously increased malformation rate and severe defects in adults, such as wrinkled wings or curved legs (Figs. 3b–d; 4). Similar phenomena were also detected in the dsXM_029863591.1-treated group (Fig. 5). Taken together, these results demonstrated that miR-989 is involved in chitin metabolism in *Ae*. *albopictus* pupae by targeting XM_029863591.1.

In addition, XM_029863591.1 was found to be a chitin-binding protein (CBP) that encodes three chitin-binding domains (CBDs) (Fig. 1d). CBPs are the major group of proteins that contain one or more CBDs, combining with chitin via CBDs and participating in the formation, maintenance and regulation of the functions of extracellular structures [39, 40]. The highest expression level was in the epidermis, suggesting that XM_029863591.1 plays a vital role in the formation of the cuticle through its interaction with chitin. It can be speculated that overexpression and suppression of miR-989 affected the transcript level of XM_029863591.1, which resulted in microstructural changes in cuticles in the epidermis. This deduction was supported by the results of HE staining, the irregular size of epidermal cells and the uneven thickness of newly formed cuticle, suggesting that miR-989 and XM_029863591.1 are required for the stability of the epidermis (Fig. 6a, b’). In particular, XM_029863591.1, a chitin-binding protein, is closely related to the formation and maintenance of the new epidermis [41, 42].

Chitin metabolism in insects is complex and dynamically regulated by numerous enzymes. Uncovering the regulatory relationships of miRNAs with chitin metabolism-related enzymes is important for elucidating the underlying molecular mechanisms of chitin metabolism and could aid in exploiting effective targets for disrupting this process. The results of this study demonstrated that overexpression of miR-989 increased and decreased the expression levels of *AaCHS*1 and *AaCht*10, respectively (Fig. 8a, b). Likewise, depletion of XM_029863591.1 resulted in an increase in *AaCHS*1 transcript level and a decrease in *AaCht*10 transcript level (Fig. 8c, d). These results demonstrated that altered expression levels of miR-989 and XM_029863591.1 can influence transcript levels of *AaCHS*1 and *AaCht*10, which might be the cause of changed chitin synthesis and degradation and lead to lethal phenotypes during the pupal-adult transition process.

Moreover, according to the evidence from HE staining and fluorescence staining of chitin, the thickened old and new cuticles in the miR-989 agomir-treated group were accompanied by an enhanced fluorescence signal of chitin. These results should be attributed to the increased expression level of *AaCHS*1 and decreased expression level of *AaCht*10 after overexpression of miR-989, which resulted in the accumulation of newly synthesized chitin and decreased degradation of old chitin. Similarly, changes in the expression levels of *AaCHS*1 and *AaCht*10 caused thickened old cuticle and enhanced the fluorescence signal of chitin in the dsXM_029863591.1-treated group (Fig. 7). All of these results demonstrated that miR-989 and XM_029863591.1 play crucial roles in chitin metabolism in *Ae*. *albopictus* and that changing their expression levels could affect the structure of cuticles and ultimately disrupt pupa development.

## Conclusion

Overall, the results of this study confirmed that miR-989 is involved in the chitin metabolism of *Ae*. *albopictus* pupae by targeting XM_029863591.1. The changes in the expression levels of miR-989 and XM_029863591.1 can obviously affect the developmental process of *Ae*. *albopictus*. Therefore, miR-989 could be utilized as an efficient target to disrupt the pupal-adult transition of *Ae*. *albopictus*. However, we are currently unable to fully explain the mechanism(s) by which miR-989 and XM_029863591.1 affected the transcript levels of *AaCHS*1 and *AaCht*10. The results of the current study provide important clues for revealing the underlying molecular mechanisms by which miRNAs participate in chitin metabolism.

### Supplementary Information

**Additional file 1: Table S1.** Primers used for qRT-PCR and RNAi. **Table S2.** Sequences of agomir and agomir NC of miR-989.

**Additional file 2:** **Figure S1.** Expression level of XM_029878882.1 after overexpression of miR-989.

## Data Availability

All relevant data are within the manuscript and its Additional files.
