# Supplementary material for: MicroRNA-989 controls Aedes albopictus pupal-adult transition process by influencing cuticle chitin metabolism in pupae
Source: Parasit Vectors. 2023 Nov 2;16:397. doi: 10.1186/s13071-023-05976-x (PMC10623821; doi:10.1186/s13071-023-05976-x)
Supplement: Supplementary file 1 — Additional file 1: Table S1. Primers used for qRT-PCR and RNAi. Table S2. Sequences of agomir and agomir NC of miR-989. [file 13071_2023_5976_MOESM1_ESM.docx]

**Table S1. Primers used for qRT-PCR and dsRNA synthesize**

| Primer name | Sequence (5'-3') |
| --- | --- |
| miR-989_F | GCGCGTGTGATGTGACGTAG |
| miR-989_R | AGTGCAGGGTCCGAGGTATT |
| miR-989_RT | GTCGTATCCAGTGCAGGGTCCGAGGTATTCGCACTGGATACGACGTACCA |
| *AaCHS*1_F | GGAGACCAAAGGATGGGACG |
| *AaCHS*1_F | GACCACGAAGAAGAGCAACACC |
| *AaCht*10_F | CTTCTACCACCACTACAACA |
| *AaCht*10_R | AGCGACTTCCTGATACGA |
| XM_029863591.1_F | GGCAGTGTCAGGGAGGAT |
| XM_029863591.1_R | CGCTGTTGCTGTGGGTTA |
| dsXM_029863591.1_F | TAATACGACTCACTATAGGGGTTTGCGAGTCGATAGGAGC |
| dsXM_029863591.1_R | TAATACGACTCACTATAGGGTTAGCGTCATCGTTGCAGAG |
| β actin_F | GCTACGTCGCCCTGGACTT |
| β actin_R | AGGAACGACGGCTGGAGA |
| U6_F | GATTAGCATGGCCCCTGC |
| U6_R | GTGCGTGTCGTGGAGTCG |
| U6_RT | GTCGTATCCAGTGCGTGTCGTGGAGTCGGCAATTGCACTGGATACGACAATATG |
| ds*eGFP*_F | TAATACGACTCACTATAGGGCAGTGCTTCAGCCGCTAC |
| ds*eGFP*_R | TAATACGACTCACTATAGGGGTTCACCTTGATGCCGTTC |

**Table S2. Sequences of agomir, and NC of miR-989**

| Type | Sequence (5'-3') |
| --- | --- |
| miR-989 agomir | UGUGAUGUGACGUAGUGGUAC |
| agomir NC | UUCUCCGAACGUGUCACGUTT |
