# Supplementary figures and images for: MicroRNA-989 controls Aedes albopictus pupal-adult transition process by influencing cuticle chitin metabolism in pupae
Source: Parasit Vectors. 2023 Nov 2;16:397. doi: 10.1186/s13071-023-05976-x (PMC10623821; doi:10.1186/s13071-023-05976-x)

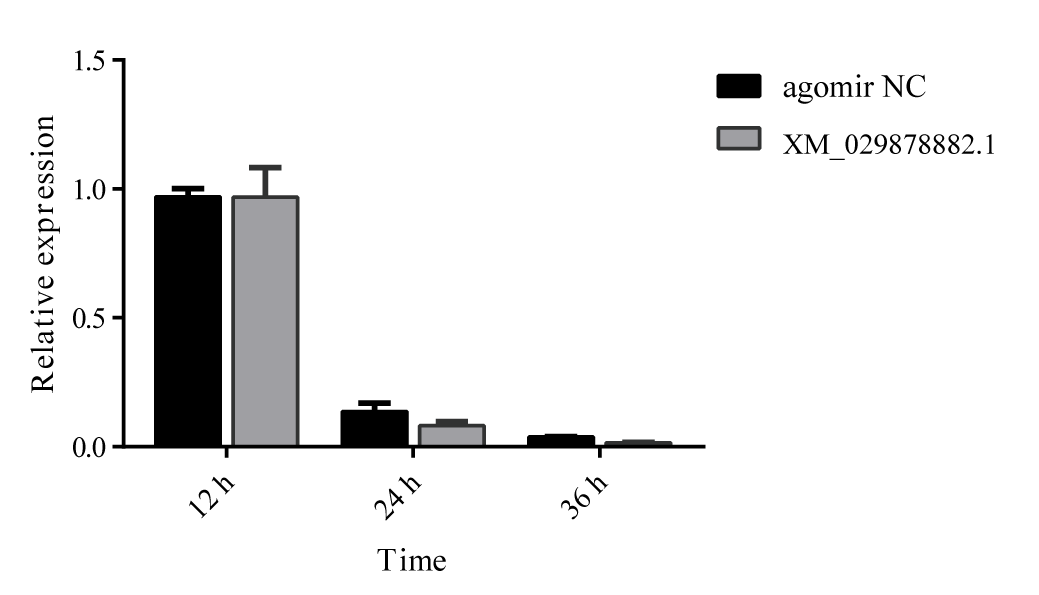

Supplement: Supplementary file 2 — Additional file 2: Figure S1. Expression level of XM_029878882.1 after overexpression of miR-989. [file 13071_2023_5976_MOESM2_ESM.tif]
